# Glucose-mediated template-free synthesis of hollow CuO microspheres†

**DOI:** 10.1039/c8ra00684a

**Published:** 2018-04-17

**Authors:** Hai Zhou, Min Kang, Biao Qin, Ning Zhao, Dong Wu, Baoliang Lv, Qingjie Wang

**Affiliations:** Department of Chemistry and Chemical Engineering, Zunyi Normal College Zunyi 563006 China km20056570@163.com +86-0851-28924799 +86-0851-28924799; Academician Workstation of Zunyi Normal College Zunyi 563006 China; State Key Laboratory of Coal Conversion, Institute of Coal Chemistry, Chinese Academy of Sciences Taiyuan 030001 China Zhaoning@sxicc.ac.cn +86-0351-4041153 +86-0351-4063121; State Key Laboratory of Advanced Chemical Power Sources Zunyi 563006 China

## Abstract

In this work, we demonstrated a facile template-free method for the preparation of hollow CuO microspheres *via* a conventional hydrothermal reaction. The hollow architecture formed directly during the hydrothermal treatment of copper nitrate and glucose, without the use of template, precipitant and calcination process. The effects of reaction time, reaction temperature and glucose concentration were investigated in detail. On the basis of experimental results, the formation of hollow CuO microspheres probably proceeded *via* self-assemble process and the subsequent Ostwald's ripening. This synthetic strategy strongly depended on the characteristics of copper nitrate, which made it could not extend to other copper salts and/or nitrates. Even though, glucose still showed efficient morphology controlling ability with respect to nanosized transitional metal oxides, which could be used for the controllable synthesis of nanomaterials.

## Abstract 2

In this work, we demonstrated a facile template-free method for the preparation of hollow CuO microspheres *via* a conventional hydrothermal reaction.

## Graphical abstract

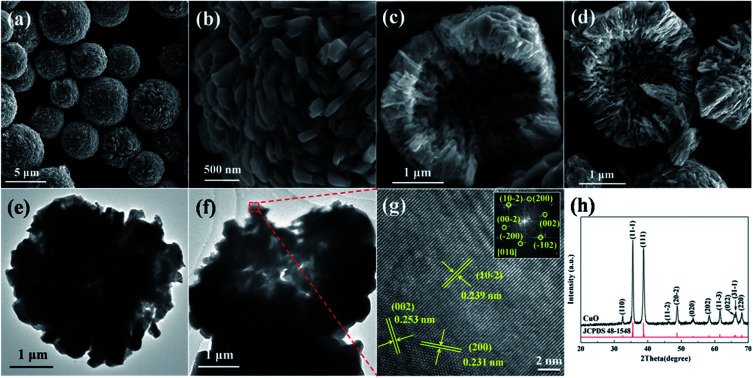

## 1 Introduction

Hollow structures have attracted increasing attention due to their variety of applications in the fields of catalysis, energy, gas sensors, drug delivery and so on. This is because the unique hollow architectures could provide enhanced surface-to-volume ratio, reduced transport length for mass and charge, accommodation space for reaction products, and volume buffering space.^1,2^ So far, hollow micro-/nano-structured materials, such as hollow spheres,^3–8^ multishelled hollow spheres,^9–11^ nanotube^12,13^ and so on have been successfully synthesized.

Several synthetic methods have been developed with the aim of creating and tailoring the size, morphology and even shell number of hollow structures. Hard templating method utilize rigid materials, such as carbonaceous spheres,^10,11^ SiO_2_ spheres^14^ and so on as template. For example, using carbonaceous microspheres as template, multiple-shell metal oxide hollow microspheres could be prepared *via* repeated templating and controlled calcination.^11^ And hollow SnO_2_ spheres with the shell thickness of about 25 nm were synthesized using SiO_2_ spheres as template, after the removal of SiO_2_, coating carbon precursor and subsequent thermal treatment, tin-nanoparticles encapsulated in hollow carbon spheres were obtained.^14^ On the other hand, soft templating method often take advantage of micelles.^9,15^ Wang *et al.* synthesized multishelled hollow Co_3_O_4_ spheres by using multilamellar PVP micelles possessing different shell numbers.^9^ Templating methods can commonly be generalized, which may be the best pathway for the preparation of complex multishelled hollow structures^2,11^.

Even though, template-free synthesis of hollow structures is desirable due to its advantages of simple and convenient. Whereas template-free synthesis requires growth units to assemble into the desired structures,^2^ thus it remains a great challenge to develop a facile template-free approach for the synthesis of well defined hollow structures. By using the well known Ostwald's ripening phenomena, hollow structures could be created *via* gradual dissolution and recrystallization, which is one of the effective methods for template-free synthesis.^16,17^ This originates from the fact that particles located in the inner cores generally possess relative higher surface energy as compared to that located in the exteriors, which is caused by the defects of mismatch generated during the initial high rate growth process. Accordingly, the inner cores would dissolute gradually leading to the formation of hollow cores.

It was widely reported that carbon spheres with metal ions incorporated into their hydrophilic shell could form between hydrothermal reaction of glucose and metal precursors.^4–6^ Hollow metal oxide spheres could be obtained after the subsequent removal of carbon *via* calcination, hollow spheres of Fe_2_O_3_, NiO, Co_3_O_4_, CeO_2_, MgO and CuO were prepared *via* this method.^6^ While in the present work, taking advantages of Ostwald's ripening process, we demonstrated a template-free synthesis of hollow CuO microspheres with the assistance of glucose *via* a conventional hydrothermal method. Unlike the results reported previously, the present hollow CuO microspheres formed directly during the hydrothermal process without the use of template, precipitant and even calcination process. We also found that this synthetic strategy strongly depended on the characteristics of copper nitrate, thus it could not be extended to other copper salts (*e.g.* CuCl_2_ and CuSO_4_) and/or other nitrates (*e.g.* Co(NO_3_)_2_, Ni(NO_3_)_2_, Fe(NO_3_)_3_ and Zn(NO_3_)_2_). Even though, we still found the efficient morphology controlling ability of glucose with respect to nanosized transitional metal oxides, which could be used for the controllable synthesis of nanomaterials. The present hollow CuO microspheres might be used in the fields of catalysis, gas sensors, superconductors, lithium ion batteries, *etc*.

## 2 Experimental

### 2.1 Synthesis of hollow CuO microspheres

All reagents were of analytical grade and used without further purification. The typical preparation process was as follows: 0.0515 g of anhydrous glucose and 0.5004 g of Cu(NO_3_)_2_·3H_2_O (the molar ratio was *ca.* 0.138) were dissolved in 80 mL distilled water under ultrasonic radiation. The as-formed solution was sealed in a Teflon-lined autoclave of 150 mL capacity, and maintained at 220 °C for 24 h. After cooling to room temperature naturally, the products were collected by centrifugation, and washed with ultrapure water three times and absolute ethanol twice to remove impurities. The final products were dried in air at 80 °C for 6 h.

In order to investigate the formation mechanism of the hollow CuO microspheres, a series of condition-dependent experiments were carried out. The reaction time was altered from 1 h to 2 h, 4 h, 8 h, 16 h and 24 h by preserving the other reaction conditions constant. The influence of reaction temperature was investigated by varying reaction temperature in the range of 160–220 °C. And the molar ratio of glucose to Cu^2+^ was varied from 0.03 to 0.05, 0.138 and 0.3 to investigate the effects of glucose concentration.

Moreover, the hydrothermal synthesis was also performed using CuCl_2_, CuSO_4_·5H_2_O, Co(NO_3_)_2_·6H_2_O, Ni(NO_3_)_2_·6H_2_O, Fe(NO_3_)_3_·9H_2_O and Zn(NO_3_)_2_·6H_2_O as metal source. The molar ratio of anhydrous glucose to these metal salts was kept at 0.138, the other reaction conditions were kept constant.

### 2.2 Characterization

The solid products were characterized by means of SEM (FEI Scios), TEM (FEI Tecnai F20) and XRD (Rigaku D/max-rB diffractometer using Cu Kα radiation, *λ* = 0.15408 nm). Thermogravimetric analysis (TGA) was performed in the temperature range of 50–600 °C under air atmosphere with a heating rate of 5 °C min^−1^ (Shimadzu DTG-60).

## 3 Results and discussion

### 3.1 Characterizations of the hollow CuO microspheres

**Fig. 1:**
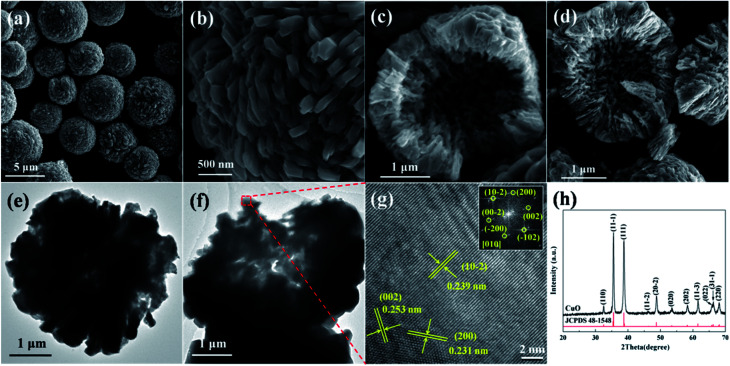
Characterizations of the hollow CuO microspheres: (a)–(d) SEM images, (e) and (f) TEM images, (g) HRTEM image and (h) XRD pattern. The inset shown in (g) is the corresponding FFT pattern.

The characterizations of the typically synthesized samples are shown in Fig. 1. As can be seen in Fig. 1a, well shaped microspheres with diameter of several micrometers were synthesized. The surface of these spheres is composed by polyhedra (Fig. 1b). The cracked spheres clearly indicate the hollow center of the microspheres, and the thickness of the shell is about one-fourth of the diameter (Fig. 1c and d). The crystallinity of the microspheres is shown in Fig. 1h. Clearly, all peaks in the XRD pattern can be perfectly indexed to CuO (JCPDS 48-1548), no characteristic peaks of any other impurities could be identified. The narrow sharp peaks suggest the high crystallization of the microspheres.

The details of the surface facets and crystallization of the as-prepared hollow CuO microspheres were also investigated by means of TEM and HRTEM. Fig. 1e and f show the TEM images of a complete and a cracked microsphere, respectively. Due to its relative large size and thick shell, the contrast between the dark edge and pale core of the sphere is not very obvious. Even though, the hollow nature of the microspheres could also be confirmed by TEM images shown in Fig. 1e and f. Fig. 1g shows the HRTEM image of the marked region in Fig. 1f. Three main kinds of lattice fringes can be identified, and the corresponding interplane distances are 0.239 nm, 0.253 nm and 0.231 nm, which respectively match the interplane distances of {10−2}, {002}, {200} and/or their equivalent facets under the incident electron beam along the [010] direction. The unambiguous lattice fringes and sharp diffraction spots of the corresponding FFT pattern reveal the well crystalline nature of the synthesized samples. Based on the above analysis, it can be concluded that hollow CuO microspheres are directly synthesized *via* the present template-free hydrothermal method.

### 3.2 Effects of synthesis conditions and formation mechanism

**Fig. 2:**
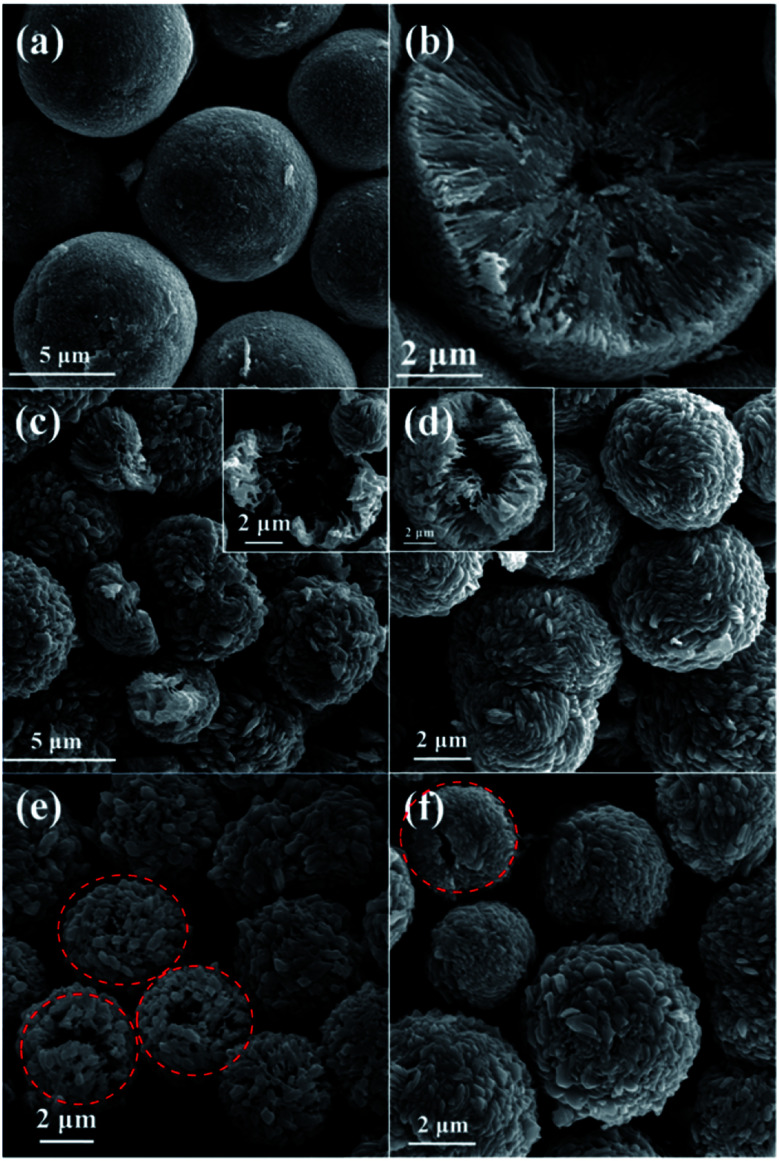
SEM images of samples prepared at the synthesis time of (a) and (b) 1 h, (c) 2 h, (d) 4 h, (e) 8 h and (f) 16 h.

In order to investigate the growth process of the hollow CuO microspheres, the effects of reaction time was varied from 1 h to 2 h, 4 h, 8 h, 16 h and 24 h, the corresponding SEM images of the obtained samples are presented in Fig. 1 and 2. As shown in Fig. 2a, well shaped spheres with relative smooth surface and diameter of *ca.* 8 μm are obtained after hydrothermal treatment for 1 h. And a relative small hollow center is generated at this stage, as shown by the cracked sphere (Fig. 2b). It should be mentioned that only trace amount of solid products could be obtained after 1 h reaction. After reaction for 2 h, much more solid samples are collected (Fig. 2c). The diameter of the spheres decreases to 5–6 μm, and polyhedra structures can be observed on the surface (Fig. 2c). Moreover, the hollow core significantly grows during this stage, leading to the thinner shell. As shown in Fig. 1 and 2(d–f), hollow spheres could be observed for all the samples prepared during the following reaction time. And the polyhedra structures on the surface of spheres become more and more apparent, while the diameter of these spheres keep almost the same (Fig. 2c–f). XRD results show that the composition of all samples prepared with the reaction time of 2–24 h are pure CuO, and extension of reaction time makes the characteristic peaks stronger (Fig. S1†).

As mentioned before, carbon spheres with metal ions incorporated into their hydrophilic shell would form *via* hydrothermal treatment by using high concentration of glucose and low concentration of metal precursors.^4–6^ Hollow metal oxide spheres could be prepared after the removal of carbon *via* calcination. While in this study, it was found that Cu(NO_3_)_2_ could directly transform into hollow CuO microspheres through hydrothermal treatment with the assistance of glucose. It is known that Cu(NO_3_)_2_ is readily to decompose.^4^ Accordingly, the formation of the present CuO is most probably accomplished *via* the decomposition of Cu(NO_3_)_2_ under hydrothermal conditions, because no precipitant and calcination process are involved. We also find that Cu(NO_3_)_2_ could even decompose directly under hydrothermal conditions without the addition of glucose. However, only little amount of irregular solid products could be obtained (Fig. S2†). Furthermore, TGA curves show that the addition of glucose does not significantly change the thermal decomposition behavior of Cu(NO_3_)_2_ under air atmosphere, the final decomposition temperature of the mixture of glucose and Cu(NO_3_)_2_ is somewhat higher than that of pure Cu(NO_3_)_2_ (Fig. S3†). As a result, glucose plays a key role for the decomposition of Cu(NO_3_)_2_ and the formation of hollow CuO microspheres under the present hydrothermal conditions.

**Fig. 3:**
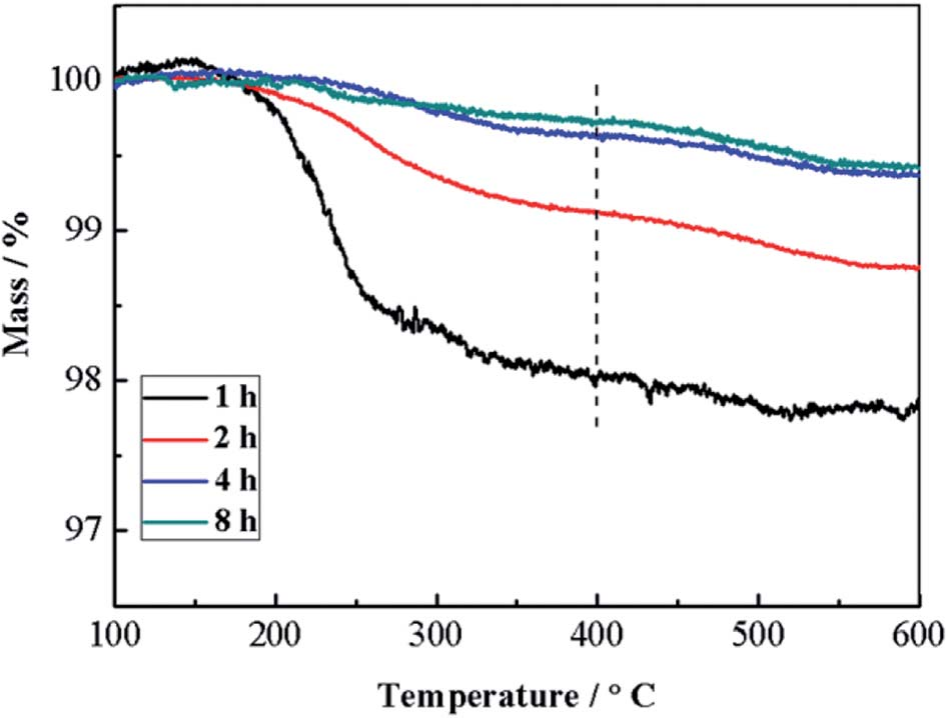
TGA curves of samples prepared under the reaction time of 1–8 h.

The solid products prepared under the reaction time of 1–8 h were also analyzed by TGA (Fig. 3). Apparently, all samples show main mass loss in the temperature range of 100–400 °C. Because only Cu(NO_3_)_2_ and glucose are used, and the composition of the products is CuO, thus the mass loss could mainly be ascribed to the decomposition of the trace amount of carbides formed *via* dehydration, decarbonylation and aromatization of glucose under hydrothermal conditions.^18,19^ Meanwhile, samples obtained after 1 h hydrothermal treatment show the greatest mass loss, and the mass loss gradually decreases with the prolongation of reaction time. Similar TGA curves are obtained based on the samples prepared with the reaction time of 4 h and 8 h. As a result, it can be confirmed that a little amount of carbides would form and present in the solid products during the initial hydrothermal treatment, and the decomposition of carbides *via* the reaction between carbides and Cu(NO_3_)_2_ mainly proceeds during the initial a few hours. Accordingly, the morphology transformation of CuO microspheres mainly proceeds during the subsequent reaction time.

**Fig. 4:**
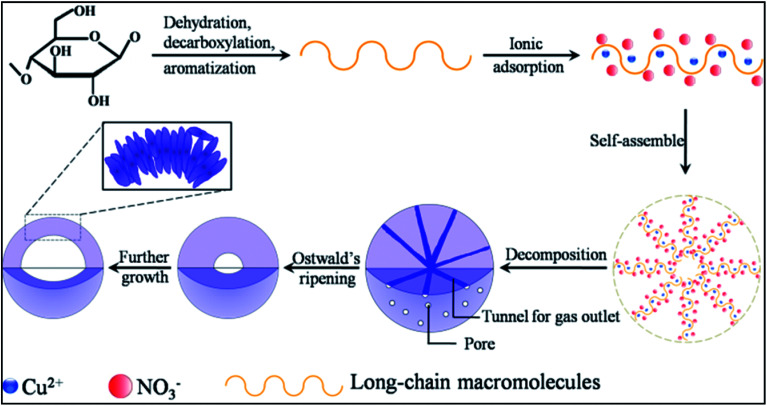
Schematic illustration of the possible formation mechanism of the present hollow CuO microspheres.

Based on the above analysis, the formation of the present hollow CuO microspheres most probably proceeds *via* self-assemble process followed by Ostwald's ripening, as illustrated in Fig. 4. Firstly, oxygen-rich long-chain macromolecules would form under high temperature hydrothermal conditions.^18^ After that, Cu^2+^ adsorbs onto the macromolecules due to the oxygen groups of these macromolecules. Macromolecules containing Cu^2+^ and NO_3_^−^ would gather together to decrease the surface energy of these intermediate species, which leads to the formation of sphere nuclei. It should be mentioned that the concentration of glucose is significantly lower than Cu(NO_3_)_2_, thus the formation of carbon spheres with the incorporation of metal ions is prohibited.^6,20,21^ In other words, the main composition of the sphere nuclei is Cu^2+^ and the charge balancing anion (NO_3_^−^). Under the high temperature hydrothermal conditions, the adsorbed Cu(NO_3_)_2_ would react with carbonaceous macromolecules, leading to the formation of CuO spheres and releasing of CO_2_ and smelly NO_2_, which could be expressed in the following pathway.Cu(NO_3_)_2_ + C_*x*_H_*y*_O_*z*_ → CuO + CO_2_ + NO_2_ + H_2_O

**Fig. 5:**
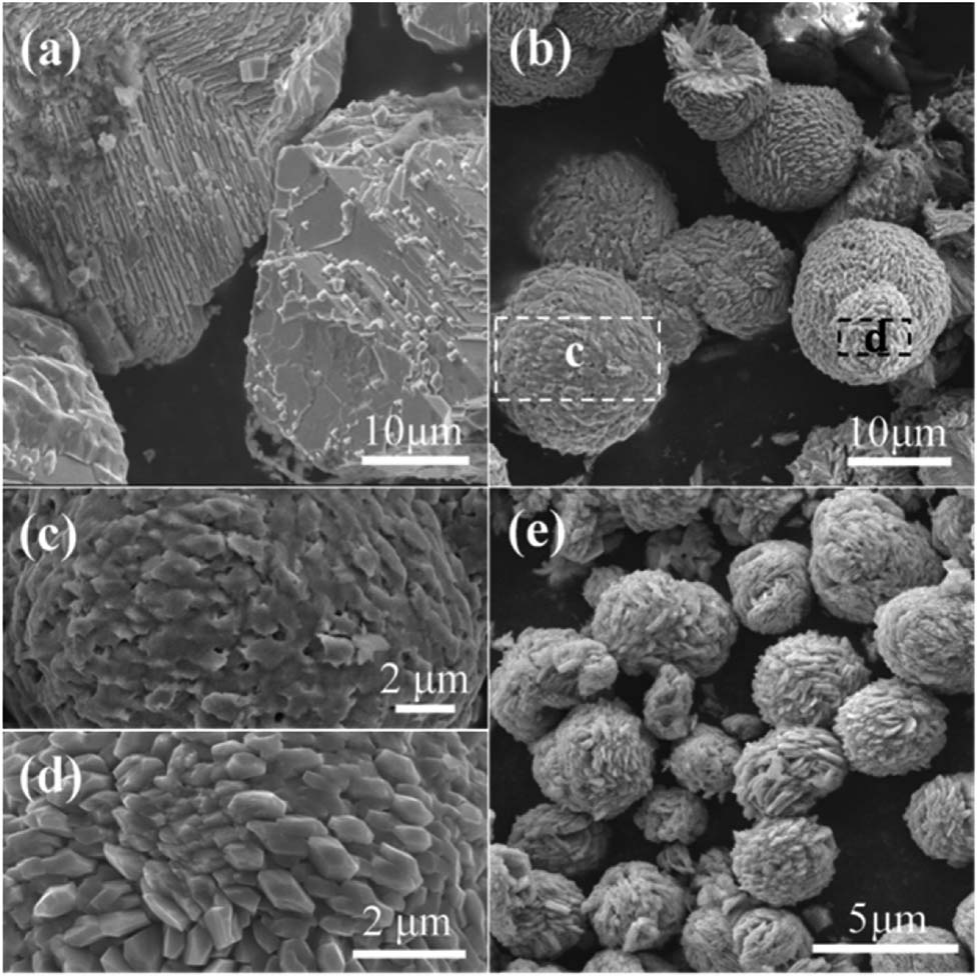
SEM images of samples prepared under the reaction temperature of (a) 160 °C, (b)–(d) 180 °C and (e) 200 °C. (c) and (d) are high magnification images of the marked regions c and d in (b).

The well known Ostwald's ripening process is driven by the energy difference between the larger particles and smaller ones, which results in the further growth of larger particles at the expense of smaller ones.^3^ Particles located in the cores generally possess smaller size and higher surface energy as compared to those in the exteriors, thus particles in the cores would dissolve gradually, and the released metal ions participate the subsequent recrystallization process. As a consequence, hollow architectures are produced *via* the gradual dissolution and recrystallization processes (Fig. 2). Moreover, the generated gases lead to the formation of pores and channels in the shell (Fig. 2 and 5), which serve as the gateway for mass exchange between the interior cavity and the exterior space during Ostwald's ripening process. As shown in Fig. 2a, spheres with hollow architecture are prepared after 1 h hydrothermal treatment, whereas no solid product could be collected when the reaction time is 0.5 h. This indicates that self-assemble process of the initial formed intermediates proceeds much faster than the subsequent Ostwald's ripening.

Meanwhile, the hydrothermal treatment was also performed using CuCl_2_ and CuSO_4_ as copper source under the same reaction conditions, as shown in Fig. S4.† Neither spheres nor CuO could be produced, which mainly due to the fact that CuCl_2_ and CuSO_4_ are more difficult to decompose as compared with Cu(NO_3_)_2_. Even though sulphate and chlorizated salts incorporated into carbonaceous spheres could decompose into metal oxides under higher temperatures (*e.g.* 550 °C).^6^

The influence of reaction temperature on the morphology of the obtained samples was also investigated by varying the reaction temperature from 160 °C to 180 °C, 200 °C and 220 °C, the results are shown in Fig. 1 and 5. It can be seen that block structures with the size larger than 10 μm are obtained under 160 °C (Fig. 5a). Increasing the reaction temperature to 180 °C, microspheres are prepared and the size of particles significantly decreases to about 10 μm (Fig. 5b). Meanwhile, spheres possessing different surface are identified in Fig. 5b, as shown by the marked regions. The sphere presented in Fig. 5c exhibits rough surface, and a large number of pores could also be observed on its surface, which may be caused by the generation of gases (mainly CO_2_ and NO_2_) during the hydrothermal process. On the other hand, the surface of sphere shown in Fig. 5d is composed of well-shaped polyhedra. When the reaction temperature is increased to 200 °C, spheres with the size of about 5 μm could be obtained (Fig. 5e). By further increasing the reaction temperature to 220 °C, well-shaped microspheres could thus be prepared.

**Fig. 6:**
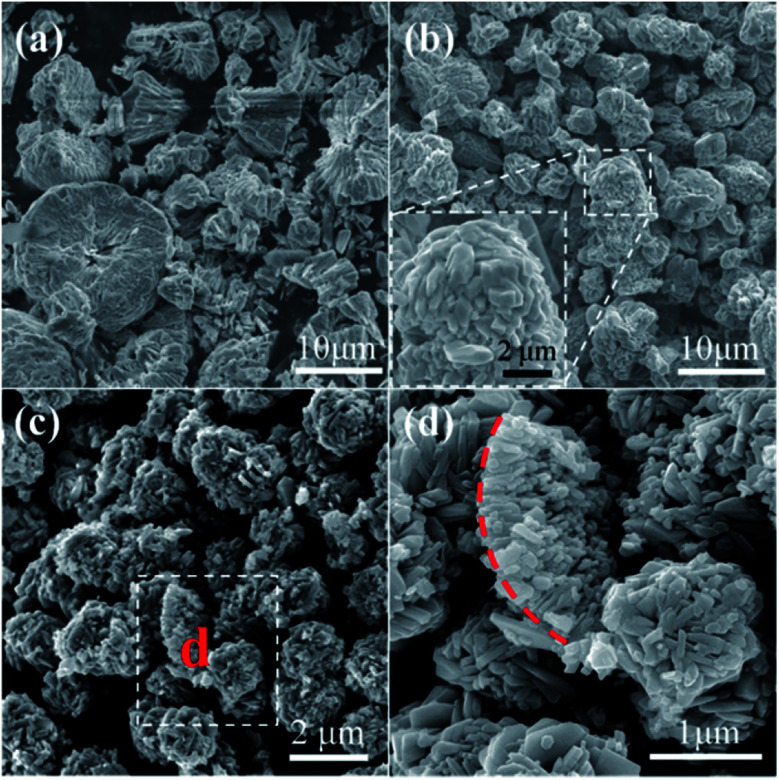
SEM images of samples prepared with different molar ratio of glucose to Cu^2+^: (a) 0.03, (b) 0.05 and (c) and (d) 0.3.

The above results suggest that the size and morphology of final samples strongly depend on the reaction temperature, *e.g.* high reaction temperature is helpful for the preparation of microspheres possessing well shape and small size distribution. This is most probably because high temperature favors the carbonization of glucose, decomposition of adsorbed Cu(NO_3_)_2_ and the subsequent Ostwald's ripening process.

The molar ratio of glucose to Cu^2+^ was then varied from 0.03 to 0.05, 0.138 and 0.3 by using 0.5004 g of Cu(NO_3_)_2_·3H_2_O while varying the loading amount of glucose, so as to investigate the influence of glucose concentration on the morphology of the final samples, as shown in Fig. 1 and 6. Solid spheres with the diameter larger than 10 μm are prepared when the molar ratio is 0.03 (Fig. 6a). It seems that the smaller particles are probably formed *via* the fracture of the spheres. Micro-sized irregular spheres possessing polyhedra on the surface and relative large size distribution are obtained under the molar ratio of 0.05 (Fig. 6b). By further increasing the molar ratio to 0.3, micro-sized porous spheres aggregated by polyhedra are prepared (Fig. 6c). Moreover, the particle marked by red dotted line is probably a portion of a sphere shell (Fig. 6d), which indicates that spheres prepared at this molar ratio probably possess hollow architecture.

The above results indicate that compact particles are prepared when the molar ratio of glucose to Cu^2+^ is smaller than the optimum value 0.138 (Fig. 6a and b). Undoubtedly, using smaller amount of glucose, more amount of Cu(NO_3_)_2_ would adsorb onto the carbonaceous macromolecules, which makes the decomposition of Cu(NO_3_)_2_ more difficult and the generation of fewer gases. Consequently, the Ostwald's ripening process is largely hindered, leading to the formation of compact particles. On the other hand, using more amount of glucose, Cu(NO_3_)_2_ could react with more amount of carbonaceous macromolecules. Accordingly, this could accelerate the decomposition of Cu(NO_3_)_2_ leading to the formation of CuO and the generation of large amount of gases, which is benefit for the subsequent dissolution and recrystallization process. As a result, porous particles composed by nanosized polyhedra are thus synthesized.

**Fig. 7:**
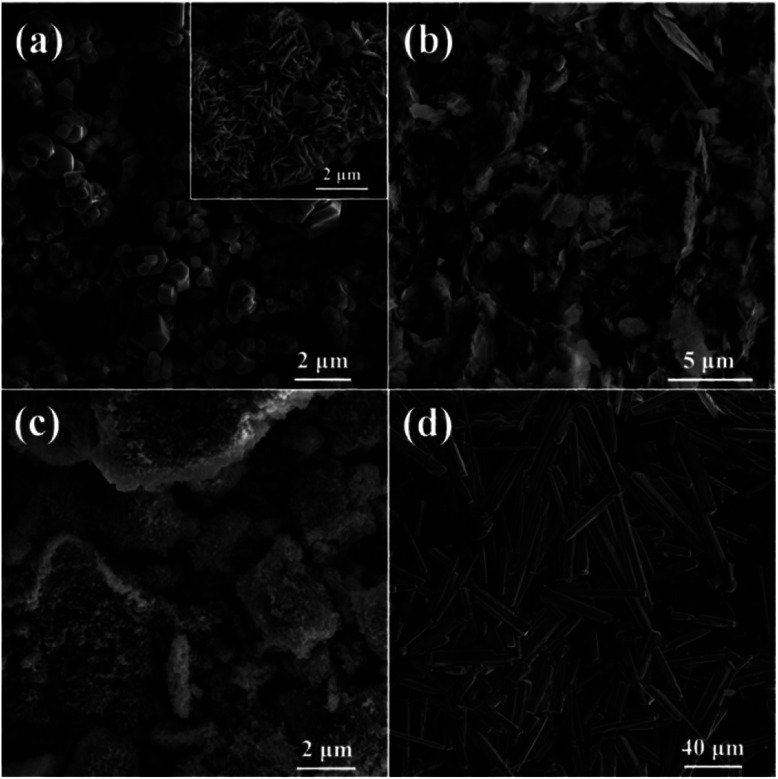
SEM images of samples prepared using (a) Co(NO_3_)_2_, (b) Ni(NO_3_)_2_, (c) Fe(NO_3_)_3_ and (d) Zn(NO_3_)_2_ as metal source.

In order to investigate the generality of this method for the preparation of other materials, we also performed the hydrothermal treatment using other nitrates as metal source under the same reaction conditions. The corresponding SEM images and XRD patterns of the samples are shown in Fig. 7 and S5,† respectively. It can be seen that nanosized Co_3_O_4_ polyhedra with some nanoplates are prepared *via* the hydrothermal treatment of Co(NO_3_)_2_ and glucose (Fig. 7a and S5a†). Ni(OH)_2_ nanoplates with a few nanoparticles are obtained using Ni(NO_3_)_2_ as metal source (Fig. 7b and S5b†). Aggregates of nanoparticles with the composition of Fe_2_O_3_ are hydrothermally synthesized using Fe(NO_3_)_3_ as metal source (Fig. 7c and S5c†). ZnO microsized rods with the length of tens of micrometers and the width of several micrometers are obtained using Zn(NO_3_)_2_ as metal source (Fig. 7d and S5d†).

Unfortunately, no sphere is prepared based on the above four nitrates, let alone hollow spheres. This indicates that the Ostwald's ripening process does not take place during the hydrothermal treatment, suggesting a totally different formation mechanism as compared with CuO. It may due to the fact that these nitrates are more difficult to decompose as compared to Cu(NO_3_)_2_, because their corresponding metals are more active than copper. Moreover, the hydrolysis properties as well as adsorption characteristics on carbonaceous macromolecules of these nitrates would greatly different from that of Cu(NO_3_)_2_. As a consequence, a significant different reaction behavior between these nitrates and glucose would be expected, as compared to Cu(NO_3_)_2_. Even though, the results shown in Fig. 7 still show efficient morphology controlling ability of glucose with respect to nanosized transitional metal oxides, which is similar to the effects of organic and/or inorganic ligands we investigated previously.^22–24^ Consequently, it is reasonable to anticipate that nanoparticles with uniform and special morphology could be synthesized *via* this facile hydrothermal method by optimizing reaction conditions.

## 4 Conclusions

A facile template-free method was demonstrated for the synthesis of hollow CuO microspheres *via* a conventional hydrothermal reaction between copper nitrate and glucose. Unlike previous reports, hollow CuO microspheres formed directly during the hydrothermal treatment without the use of precipitant and calcinations process. The effects of reaction time, reaction temperature and glucose concentration were investigated in detail. The results showed that the formation of hollow CuO microspheres probably proceeded *via* self-assemble process and the subsequent Ostwald's ripening. Although this method was not applicable for the synthesis of hollow architecture based on other metal oxides, we believed that the results reported in this work might provide some new insights into the controllable synthesis of nanomaterials with the assistance of glucose, due to its efficient morphology controlling ability.

## Conflicts of interest

The authors declare no conflicts of interest.

## Supplementary Material

RA-008-C8RA00684A-s001
