# Supplementary material for: Glucose-mediated template-free synthesis of hollow CuO microspheres
Source: RSC Adv. 2018 Apr 17;8(26):14157–63. doi: 10.1039/c8ra00684a (PMC9079924; doi:10.1039/c8ra00684a)
Supplement: RA-008-C8RA00684A-s001 [file RA-008-C8RA00684A-s001.pdf]

Electronic Supplementary Material (ESI) for RSC Advances.
This journal is © The Royal Society of Chemistry 2018

Supplementary information for

Glucose-mediated template-free synthesis of hollow CuO microspheres

Hai Zhou,[ab] Min Kang,*[ab] Biao Qin,[ab] Ning Zhao,*[c] Dong Wu,[c] Baoliang Lv[c] and Qingjie Wang[d]

[a]*Department of Chemistry and Chemical Engineering, Zunyi Normal College, Zunyi 563006, China*

[b]*Academician workstation of Zunyi Normal College, Zunyi 563006, China*

[c]*State Key Laboratory of Coal Conversion, Institute of Coal Chemistry, Chinese Academy of Sciences, Taiyuan 030001, China*

[d]*State Key Laboratory of Advanced Chemical Power Sources, Zunyi 563006, China*

*Corresponding author. Tel: +86-0851-28924799; Fax: +86-0851-28924799;

E-mail: km20056570@163.com

*Co-Corresponding author. Tel: +86-0351-4063121; Fax: +86-0351-4041153;

E-mail: zhaoning@sxicc.ac.cn

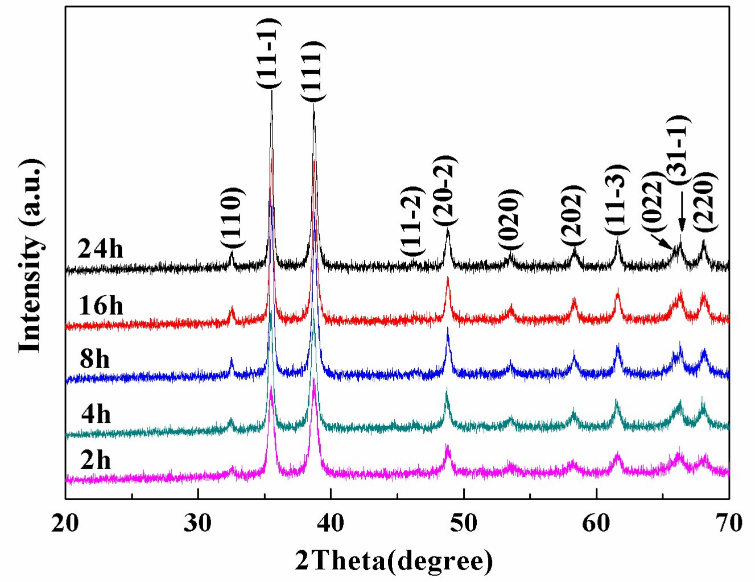

Fig. S1 XRD patterns of samples prepared at different synthesis time. Because only trace amount of sample could be obtained when the reaction time is 1 h, thus the XRD pattern of this sample is not recorded.

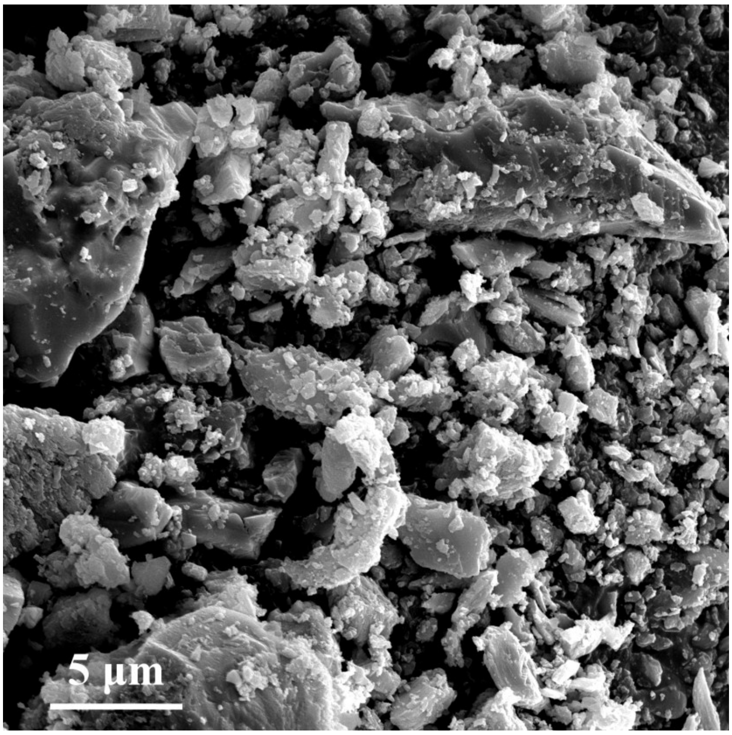

Fig. S2 SEM image of the sample obtained via the direct hydrothermal treatment of $Cu(NO_3)_2$.

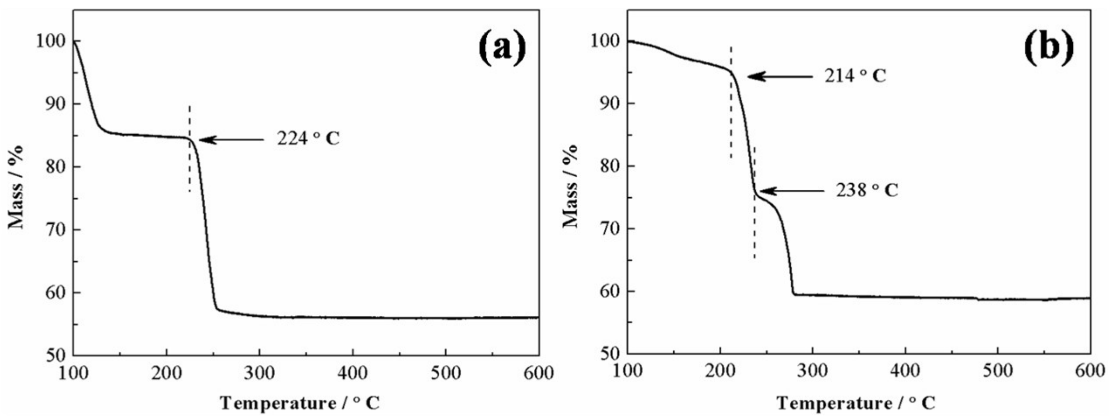

Fig. S3 TGA curves of (a) pure $Cu(NO_3)_2$ and (b) the mixture of glucose and $Cu(NO_3)_2$ under air atmosphere. The molar ratio of anhydrous glucose to $Cu(NO_3)_2 \cdot 3H_2O$ is kept at 0.138.

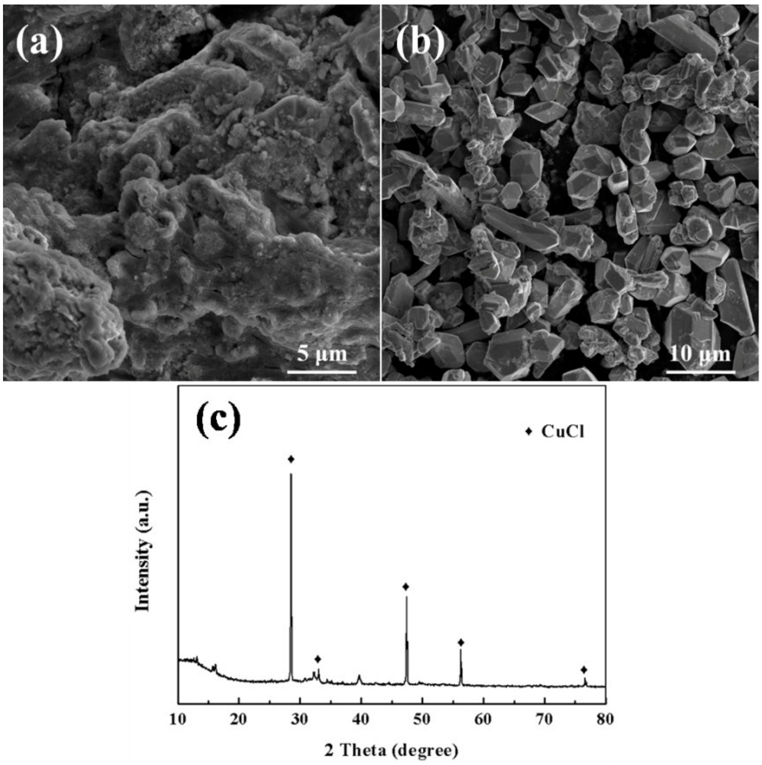

Fig. S4 SEM images of samples obtained using (a) $CuCl_2$ and (b) $CuSO_4{\cdot}5H_2O$ as copper source, (c) XRD pattern of (a).

0.0515 g of glucose was also hydrothermally treated with 0.2774 g of $CuCl_2$ and 0.5175 g of $CuSO_4{\cdot}5H_2O$ (the corresponding molar ratio is kept at 0.138), respectively. The other reaction conditions were maintained constant. Apparently, the two samples show great difference in morphology as compared to the samples prepared using $Cu(NO_3)_2$ as copper source. More importantly, CuCl but not CuO is prepared via the hydrothermal reaction between glucose and $CuCl_2$, and some unknown impurities could also be identified. The formation of CuCl suggests the reduction capacity of glucose. On the other hand, only trace amount of sample could be obtained by using $CuSO_4$ as copper source, the corresponding XRD pattern is not recorded. Even though, it could be concluded that different reaction mechanism occurs during the hydrothermal treatment using $CuCl_2$ and $CuSO_4$ as metal source, as compared with $Cu(NO_3)_2$.

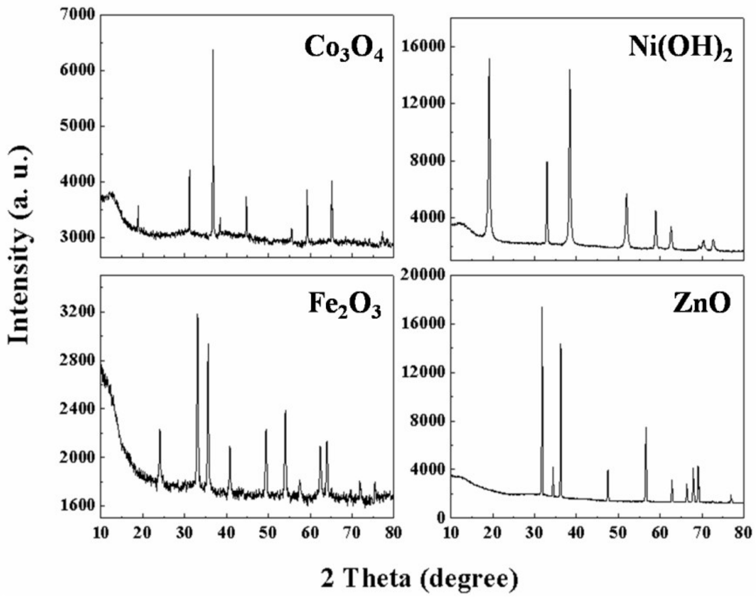

Fig. S5 XRD patterns of samples prepared using (a) $Co(NO_3)_2$, (b) $Ni(NO_3)_2$, (c) $Fe(NO_3)_3$ and (d) $Zn(NO_3)_2$ as metal source.
